# Quality science from quality measurement: The role of measurement type with respect to replication and effect size magnitude in psychological research

**DOI:** 10.1371/journal.pone.0192808

**Published:** 2018-02-12

**Authors:** Diana E. Kornbrot, Richard Wiseman, George J. Georgiou

**Affiliations:** University of Hertfordshire, Department of Psychology, University of Hertfordshire, College Lane, Hatfield, Hertfordshire, United Kingdom; Institut Català de Paleoecologia Humana i Evolució Social (IPHES), SPAIN

## Abstract

The quality of psychological studies is currently a major concern. The Many Labs Project (MLP) and the Open-Science-Collaboration (OSC) have collected key data on replicability and statistical effect sizes. We build on this work by investigating the role played by three measurement types: *ratings*, *proportions and unbounded* (measures without conceptual upper limits, e.g. time). Both replicability and effect sizes are dependent on the amount of variability due to extraneous factors. We predicted that the role of such extraneous factors might depend on measurement type, and would be greatest for ratings, intermediate for proportions and least for unbounded. Our results support this conjecture. OSC replication rates for unbounded, 43% and proportion 40% combined are reliably higher than those for ratings at 20% (effect size, *w* = .20). MLP replication rates for the original studies are: proportion = .74, ratings = .40 (effect size w = .33). Original effect sizes (Cohen’s *d*) are highest for: unbounded OSC cognitive = 1.45, OSC social = .90); next for proportions (OSC cognitive = 1.01, OSC social = .84, MLP = .82); and lowest for ratings (OSC social = .64, MLP = .31). These findings are of key importance to scientific methodology and design, even if the reasons for their occurrence are still at the level of conjecture.

## Introduction

There has been much recent concern about the reproducibility of research in science (see, e.g., [1–3]. In psychology this has led to two major replication studies. The Open Science Collaboration (OSC) attempted to replicate 100 cognitive and social psychological effects from prestige cognitive, social and general psychological journals (2). The OSC attempted one replication of each of 98 originally significant effects. The Many Labs Project [4] made 36 attempts to replicate 16 effects from 13 studies, mostly in social psychology journals (1). There were 36% of OSC and 72% of MLP effects that replicated (where an effect is defined as replicable when both an original study and its replication are significant at the 95% significance level). Using Cohen’s *d* as a common measure of effect size (the *r*—values reported by OSC were converted to *d*, using equation *d* = 2*r*/√ (1-*r*^2^)) [5], the OSC replications yielded an overall mean of .96 (.83, 1.09) whilst the studies involved in the MLP resulted in a mean of .85 (.45, 1.28).

Although the results of these studies have generated a great deal of discussion (see, e.g., 6), none of this work has focused on the relationship between the effect size, replication rates and type of the response variables used in the individual studies.

We reviewed the studies involved in the OSC and MLP, and identified three types of measures: *unbounded*, *proportion* and *ratings*. Unbounded measures have no conceptual upper limit. In the OSC and MLP databases, Unbounded measures included time, distance, physiological activity, and the signal detectability measure of discriminability, d’ (one contingency judgment study with a response scale from -100 to +100 was also included as the range was so large). Unbounded measures were defined on either the real line—∞ to + ∞, or the positive real line 0 to + ∞.

Proportion measures are defined on the interval 0,1 and include, for example, the number correct out of a fixed number of problems, or number of participants improved out of number given a treatment. Proportion measures in the OSC and MLP databases mostly used accuracy as proportion of errors (or equivalently error rate) and also some probability estimate studies.

Finally, rating measures are intrinsically ordinal, and comprise ordered verbal labels or a small number of integers with verbal labels at either end for a single item, or sums of such items. Measures in the OSC and MLP databases comprised either single Likert scales using between 1 and 11 categories, or sums of up to 12 Likert items.

For the MLP, only proportion and rating measures were considered, as four out of five studies with unbounded measures analyzed rank transformed rather than raw measure.

Supporting information, S1 Dataset, provides classification of each study and web addresses for original and replication studies.

There is good reason to expect replicability and effect size to be related to measurement type. In the OSC and MLP studies there is, typically, a response variable and one or more predictor variable(s) with single participants as the unit being measured. Statistical analyses then depend on obtaining the means, and variances, of the lowest level cell of the design. Any comparison has variability due to the predictors and to *extraneous* variability. Thus differences in extraneous variability will lead to differences in effect size and replicability. This may, in part, explain the superior performance of cognitive over social psychology for the OSC studies, as variables of interest to social psychologists tend to be more culture dependent than those of interest to cognitive psychologists.

For unbounded measures, such as clock time, there is effectively no extraneous variability due to the instrument of measurement. In contrast, for. there is binomial variability in estimating the proportion for each participant in each cell of the design. Even if the underlying mechanism that generates a proportion (e.g. proportion correct) is constant for a given person in a given cell, the measured proportion will have binomial variability in the same way that an unbiased die will not always produce one six in every six throws. The problem is worse for ratings, as the underlying mechanisms are presumed to generate an ordered multinomial distribution. It is well known from measurement and psychometrics that using rating scales especially those with few points, loses information and is less precise than continuous scale because of the coarse categorization [6].

In addition, proportion measures might be less accurate than unbounded measures, because they are subject to ceiling and floor effects (for example, if accuracy on a test is near 100%, there is little room for improvement).. are also known to have large standard error for small Ns. Ratings may be subject to additional variability due to the different ways in which diverse people interpret the verbal scales. Finally, OSC and MLP studies using ratings differed from those using proportions or unbounded (metric measures) in that only one measurement was taken per person per cell in these studies. Investigators may believe that it does not make sense to have someone rate identical scenarios more than once. By contrasts, unbounded measure of time, etc. are based on some central tendency measure of several replications for identical stimuli for the same person in any given cell; and proportion measures are by definition the frequency of some response divided by the number of replication opportunities. The number of replications per participant per cell in unbounded and proportion studies varied from 4 to 200. These possible reasons for differences in extraneous variation are presented to explain the motivation for systematically investigating measure type. None are tested or testable from these data sets.

On the basis of these diverse arguments we predicted that *rating* measures would have more extraneous variation, and hence have poorer quality performance than either proportion or unbounded measures; and that proportion would have poorer performance than unbounded measures.

A*s* the OSC found that cognitive studies outperformed social psychological studies, we also investigated whether this superiority could be explained, wholly or partly, by differential use of different measure types. Finally, we explored whether quality as assessed by authors of original OSC studies, reported by Gilbert et al. [7] was related to measure type.

## Materials and methods

### Materials

Data for the 98 OSC studies with significant original results were downloaded from the OSC supplementary material [8]. Data for up to 36 replications of 16 MLP studies [4] were downloaded from their supplementary material at https://osf.io/ydpbf/files/. Each study was then classified as to measure type (unbounded, proportion, rating) and objectivity (*objective*, *subjective*). OSC files were also categorized as to whether the original authors considered their study to be high quality (true, false) [7] in addition to the OSC’s categorization as social or cognitive. Raw data are provided in the supporting information S1 Dataset

### Analysis methods

All inferential tests were conducted at 95% significance level. Two main response variables were used. The first was replicability, pR, the proportion of studies that had null hypotheses p-values < = .05 for the original study that also had null hypotheses p-values < = .05 for the replication. The second was the Cohen’s *d* effects size.

#### Analysis of replicability, pR

Hypotheses about directional pairwise comparisons used 1-tailed Fisher’s exact tests with *w* as effect size for the replicability response variable, pR. For the OSC the following planned comparisons were made: unbounded v proportion; proportion v rating; unbounded v rating; and metric (unbounded and proportion combined) v rating. For the MLP, replication rates *pR* for each of the 36 replications were used, 562 studies in all. (One replication site had equipment failure on some studies; and for the US Flag study, International replicators were excluded, hence number of replications is less than 16 times 36). Comparison was made between proportion and rating as four out of five unbounded studies used ranked rather than raw data.

#### Analysis of effect size, Cohen’s *d*

For OSC, Cohen’s d values were calculated from published values of r, using **equation** d = 2r/√ (1-r^2^). For MLP data Cohen’s d values were taken direct from the manuscript

Planned comparisons for the response measure Cohen’s *d* were conducted, using unequal variance t-tests: for OSC cognitive (original, replication), unbounded v proportion; for OSC social (original, replication) unbounded v proportion and proportion v ratings: for MLP proportion v ratings. For the OSC, factorial analyses were conducted with Cohen’s *d* as response and with predictors: measure type (unbounded, proportion, rating) and discipline (cognitive, social) as between factors and occasion (original, replication) as a within factor. This is not a full factorial analysis as cognitive studies did not use rating measures, However it makes full use of all the available information that includes both discipline and measure type. As no cognitive studies used rating measures, separate analyses for social and cognitive were conducted with occasion and measure type (2 for cognitive, 3 for social) as predictors.

#### Power

Using G* [9], a priori power to detect a medium effect size at 95% significance level is greater than .99 for MLP for both replication and effect size. For OSC replication power is .84 for unbounded and proportion combined compared with ratings, but down to .63 for proportion v ratings and .72 for proportion v unbounded. For OSC broken down by discipline as well as measure type, power for pairwise comparisons is relatively low: for medium effects between .35 and .48 and for large effect sizes between .64 and .81. All comparisons are planned so there is no correction for multiple testing. For the 3-way ANOVA, power to detect a medium effect for the main effects and two-way interactions were above .7 with correlations .5 or less.

#### Quality assessment

For the OSC a contingency test was conducted comparing quality assessment with replicability cited by [7].

## Results

### Replicability

Table 1 summarizes the replication rates and inferential tests for the replicability measure, *pR*. For OSC, the replication rates for unbounded, 43% and proportion 40% combined are reliably higher than those for ratings at 20%, Fisher’s exact test gives p = .042, with an effect size, *w* = .20 from the Likelihood Ratio chi-square, equivalent to Cohen’s *d* = .41, a small to medium effect. For the MLP, the proportion replication rate is 74% compared to 40% for ratings. The effect for proportion v ratings *gives* w = .33 equivalent to Cohen’s *d* = .70.

**Table 1:** Summary statistics for replication rate proportion, pR.

Data Set	Measure Type	N	Mean	LCL	UCL	Comparison			
						1	2	w	Fisher Exact
OSC	U: unbounded	42	.43	.29	.58	U, P	R	.20	.042
	P: proportion	30	.40	.24	.58	P	R	.23	.074
	R: rating	25	.20	.09	.40	U	R	.23	.059
MLP	P: proportion	144	.74	.66	.80	P	R	.33	< .0005
	R: rating	239	.40	.34	.47				

Notes. LCL is lower 95% confidence level, and UCL is upper 95% confidence level. Effect size, w = √(χ^2^/N). Fisher exact is probability of null, 1-tailed.

### Effect size Cohen’s *d*

**Fig 1:**
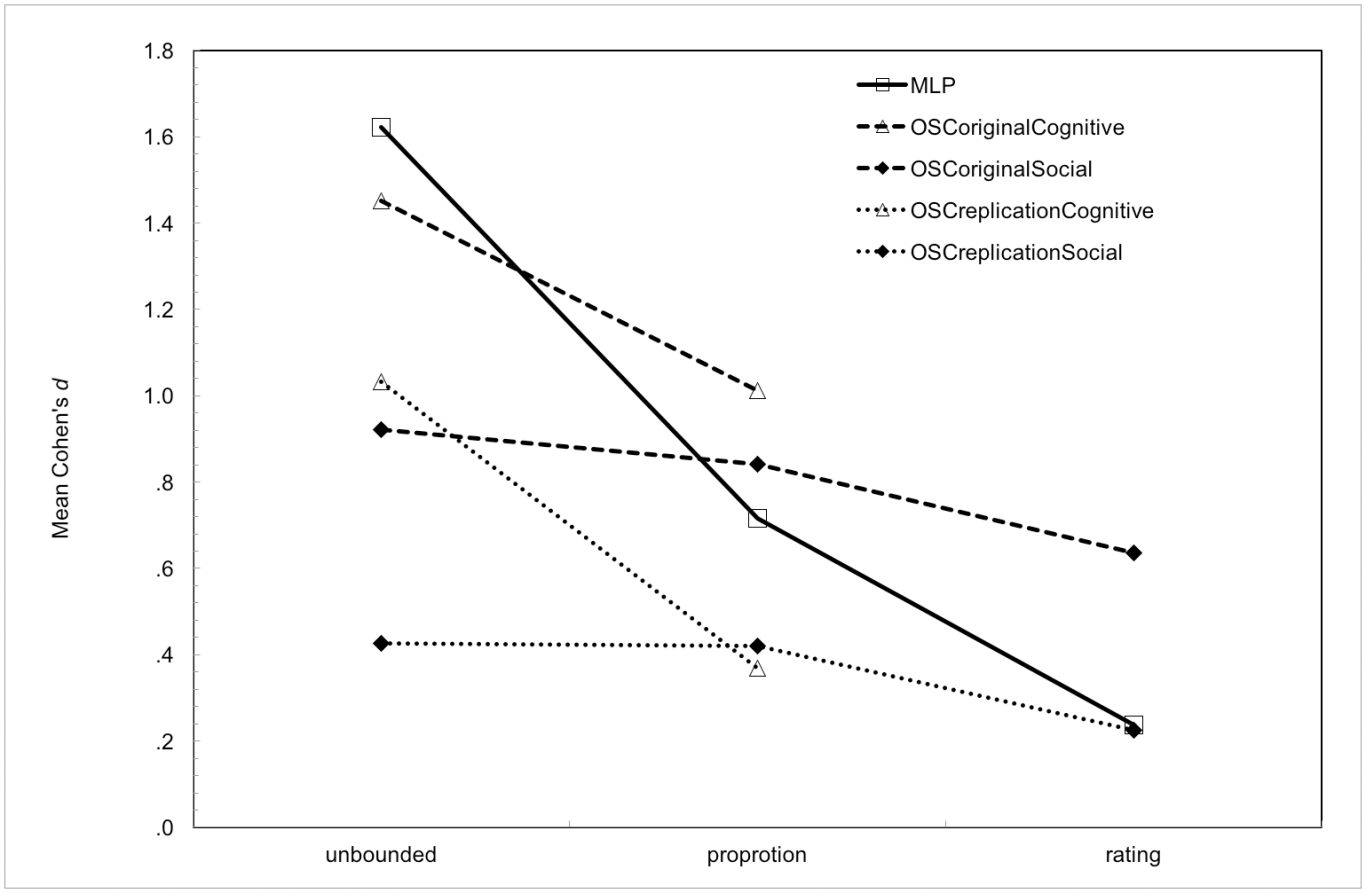
Cohen’s *d* as function of measure type for OSC cognitive, OSC social and MLP studies.

The pattern of association between measure type and effect size, Cohen’s *d* is shown in Fig 1 for: OSC cognitive, OSC social, both original and replication, and MLP studies.

Table 2 gives summary statistics for all relevant pairwise comparisons, using unequal variance t-tests, for the Cohen’s *d* effect sizes shown in Fig 1. The 3 way ANOVA with Cohen’s *d* as response and occasion (original, replication) as a repeated predictor and measure type (unbounded, proportion, ratings) and discipline (cognitive, social) as between predictors gave significant effects for occasion *F* (1, 85) = 43.9, *p* < .0005, η^2^ = .34 (original d > replication d); measure type, F (2, 85) = 3.6, p = .031 (unbounded d > proportion d > rating d), η^2^ = .08; and discipline *F* (1,85) = 3.4, *p* = .036, η^2^ = .04 (cognitive d > social d); with the discipline by measure type interaction (difference between unbounded and proportion larger for cognitive than social) just failing to reach the 95% criterion, F (1, 85) = 2.9, p = .093, η^2^ = .031. As may be seen in Fig 1 and Table 2, post hoc the interaction suggests a strong effect of measure type for cognitive studies and a non-significant effect for social studies. All interactions with replication were far from significant, p > .10. The 2-way ANOVA for social studies had main effect of replication, F (1,47) = 52.0 p < .0005, η^2^ = .53; but no main effect of measure type F (2,47) = 1.29, p = .290. Cognitive studies showed main effects of replication, F (1, 38) = 13.6, p = .001, η^2^ = .26 and of measure type, F(1,38) = 9.0, p = .005 η^2^ = .19. Interactions were non-significant for both disciplines.

**Table 2:** Summary of pairwise comparison of effect size Cohen’s *d* according to measure type and discipline.

Comparison	Data	Δ	t	df	p-null, 1 tail
Unbounded v Proportion	OSC original cognitive	.73	2.3	39.2	.**014**
	OSC replication cognitive	.78	2.5	39.4	.**009**
	OSC original social	.10	.2	26.6	.404
	OSC replication social	.01	.0	21.0	.492
Proportion v Ratings	OSC original social	.71	1.3	14.4	.100
	OSC replication social	.43	.8	13.9	.216
	MLP	.82	7.8	186.7	< .**0005**
Cognitive v Social	OSC original unbounded	.87	2.6	35.9	.**007**
	OSC replication unbounded	.69	-2.2	39.0	.**037**
	OSC original proportion	.36	.9	24.4	.193
	OSC replication proportion	-.09	-.2	19.7	.426

Notes. Effects significant at 95% level in bold.

Δ is the difference between Cohen’s *d* values for specified comparison

### Quality assessment

Contingency table analysis of the association between researchers’ quality assessment and replication showed that unbounded measures had a higher proportion of ‘good’ assessments (p_g_), p_g_ = .62, than did proportion measures, p_g_ = .56, or *rating* measures, p_g_ = .17, Likelihood Ratio χ^2^ (2) = 13.8, p = .001, w = .39.

## Discussion

As predicted, for the MLP replication, proportion measures are superior to *rating* measures for replicability, *d* = .80. For the OSC replication, with weaker power, but a more numerous and diverse range of studies, there are also clear effects of measure type. Rating measures are inferior to proportion and unbounded measures combined for replicability, with a moderate effect size of Cohen’s *d* = .47.

Overall for effect size, Cohen’s *d*, unbounded outperforms proportion, which outperforms ratings. As is evident in Fig 1 and Table 2 effect sizes are moderate to large for unbounded v proportion for OSC cognitive (replication *d* = .78, original *d* = .73); and large for MLP for proportion v ratings (*d* = .82.). No significant measure type effects were observed for OSC social.

As in previous analyses, there were significant discipline effects showing a larger *d* value for cognitive than for social studies. However this effect was confined to unbounded measures (*d* = .69 replications, *d* = .87 originals), moderate to large effects. Discipline effects were not significant for proportion or *rating* measures (although power was not high). There is insufficient data here to fully disentangle the relation between discipline and measure type. What is clear is that one observes measure type effects within discipline, OSC unbounded v proportion; and proportion v *rating* for MLP (where studies are purportedly all social).

Discipline effects are important as they suggest that variables typically used by social psychologists tend to have more extraneous variability, and/or less cultural universality than those used by cognitive psychologists. This is an observation not a criticism, as it may be an inevitable consequence of the issues studied by social psychologists.

It should be emphasized that our findings do not imply that rating measures are an inferior choice. There are many situations, cognitive as well as social, where people cannot use scales with more than 7 or so points effectively [10], and in any case prefer shorter scales. Examples include, quality ratings in competitions such as diving, gymnastics and pain. What the findings do imply is that because there is more variability, such studies may need more participants and/or more judgments per participant.

The MLP studies were non-randomly chosen as being ‘important’, and were few in number. Consequently the 11 MLP studies were less diverse than the 98 OSC studies, chosen randomly from three highly rated journals. However, the MLP performed many replications of each study thus obtaining greater accuracy for their measures of replication and effect size. The main evidence for the effect of measure type comes from the OSC, but the MLP provides important support, specifically for studies in the social domain.

Researchers’ evaluation of the quality of replication protocols was also lower for studies using *rating* measures [7]. This may have been because researchers using these measures are aware of just how difficult they are to implement,

Proportion and unbounded measures include both subjective measures and objective measures. So it might be though that the lower effect size for ratings is due to their being subjective. We checked the magnitude of differences due to classification of response measures as objective or subjective for both replicability and effect size. No differences were significant (although power was low).

The current convention for the categorization of variables (ratio, interval, ordinal, categorical) was developed by Stevens [11]. This categorization, in spite of its known limitations, is extremely prevalent (if not universal) in statistics texts for non-statistician scientists, with unbounded and proportion measures are lumped together as ‘interval’ or ‘ratio’, and ratings declared as ‘ordinal’. Our analyses suggest a fundamental re-examination of Stevens’ system, with a more nuanced approach. Indeed, unbounded, proportion, *rating* is not an exhaustive list of possible measure types. There are also analogue and bounded scales, with a large but bounded number of points: e.g. Borg scales [12], some cognitive scales. The distinction between unbounded and proportion measures matters because they have different distributional properties. These were not appreciated by Stevens, and have been mostly ignored in courses on ‘statistics for scientists’ ever since. In our view this needs to change. Generally, the categorization of variable types has been for the purpose of deciding what statistical analyses are capable of answering what substantive scientific questions. Stevens believed that knowing the arithmetic properties of variables was sufficient. He, like several of the authors of the studies discussed here, did not consider either distributional features, such as normality, heterogeneity of variance, distribution; or design features such as whether predictors are random rather than fixed. Ignoring such features means ignoring additional possible sources of variation, and these are likely to have more effect for ratings than proportion and more effect for unbounded than proportion. In our view, this is shown compellingly in our results, even though we cannot unambiguously pinpoint the exact statistical problem.

These findings have strong implications for the design of studies in psychological and other sciences. Findings can only be considered robust if they are replicable, and we have demonstrated a relationship between measurement type and replication rates. It seems likely that researchers can boost their replication rates by trying to develop unbounded measures. However, the relatively poor performance of *rating* measures does NOT imply that they should be avoided, as they are often the only way of assessing important psychological concepts.

A further implication is that researchers may need to pay more than lip service to effect sizes. Although current recommendations and practice is to provide effect sizes, their magnitude is rarely emphasized and may have little influence on evaluation of importance, see for example [13]. As some corroboration, the OSC obtained, among many other measures, ratings of “exciting”, but they report these were not significantly related to effect size. Original effect sizes for ratings were medium ~ .65 and so it was not unreasonable to publish, although replication was much lower ~ .24. Perhaps the bar for what is considered scientifically ‘important’ needs to be raised, nearer d = .8, to allow for the finding that replications are likely to have a substantially lower effect size, especially when based on relatively small N. Perhaps file drawers (with unpublished or unsubmitted studies) should be populated by low or even medium, effect size significant studies rather than high power non-significant ones.

In conclusion, measure type matters for both robustness and relevance. The OSC and the MLP have done a great service to science in providing this data. Researchers from all disciplines need to take care to use measures that generate effects of a magnitude that are important theoretically and practically.

## Supporting information

S1 Dataset This provides data sets obtained from OSC and MLP. (XLSX)
